# SimRNAweb: a web server for RNA 3D structure modeling with optional restraints

**DOI:** 10.1093/nar/gkw279

**Published:** 2016-04-19

**Authors:** Marcin Magnus, Michał J. Boniecki, Wayne Dawson, Janusz M. Bujnicki

**Affiliations:** 1 Laboratory of Bioinformatics and Protein Engineering, International Institute of Molecular and Cell Biology in Warsaw, ul. Ks. Trojdena 4, PL-02-109 Warsaw, Poland; 2 Bioinformatics Laboratory, Institute of Molecular Biology and Biotechnology, Faculty of Biology, Adam Mickiewicz University, ul. Umultowska 89, PL-61-614 Poznan, Poland

## Abstract

RNA function in many biological processes depends on the formation of three-dimensional (3D) structures. However, RNA structure is difficult to determine experimentally, which has prompted the development of predictive computational methods. Here, we introduce a user-friendly online interface for modeling RNA 3D structures using SimRNA, a method that uses a coarse-grained representation of RNA molecules, utilizes the Monte Carlo method to sample the conformational space, and relies on a statistical potential to describe the interactions in the folding process. SimRNAweb makes SimRNA accessible to users who do not normally use high performance computational facilities or are unfamiliar with using the command line tools. The simplest input consists of an RNA sequence to fold RNA de novo. Alternatively, a user can provide a 3D structure in the PDB format, for instance a preliminary model built with some other technique, to jump-start the modeling close to the expected final outcome. The user can optionally provide secondary structure and distance restraints, and can freeze a part of the starting 3D structure. SimRNAweb can be used to model single RNA sequences and RNA-RNA complexes (up to 52 chains). The webserver is available at http://genesilico.pl/SimRNAweb.

## INTRODUCTION

The ribonucleic acid (RNA) plays fundamental roles in biology, including the transmission of genetic information, regulation of gene expression and catalysis of biochemical reactions (1). Many RNA molecules or their parts (domains or motifs) fold into stable three-dimensional (3D) structures that define, at least partially, their ability to interact with other molecules and carry out their tasks within the cell (2). However, experimental determination of RNA 3D structures is laborious and challenging, and the majority of known RNAs remain structurally uncharacterized. Although a limited number of experimentally obtained RNA 3D structures are available in the Protein Data Bank (3), there are a multitude of RNA sequences [e.g. in the RNAcentral database (4)] for which no 3D structure exists. Experimental information about RNA secondary structure (base pairing pattern) has been accumulating at increasing speed, thanks to the introduction of high-throughput techniques (5,6). However, 2D diagrams that describe base-pairing interactions alone provide only part of the story. Often, one must look at the 3D structure to gain a better appreciation for the mutual position of chemical groups that may be functionally relevant and interact with other molecules. This additional information about the 3D RNA structure is neither obvious nor intuitive from the sequence or secondary structure alone.

To address the problem of the paucity of 3D structural information, computational structure prediction methods have been developing that either utilize information derived from known structures of other RNA molecules, by way of template-based modeling, or attempt to simulate the physical process of RNA structure formation, by way of template-free modeling (7–9). Some methods work on an all atom representation of the RNA molecule, while others simplify it by coarse-graining (10). These methods have a variety of strengths in certain areas that make them useful in contemplating the structural basis of biological function. For instance, there are a number of approaches that enable 3D structure prediction starting from RNA sequence alone. Some of these methods also provide a web service such as RNAComposer (11), Vfold3D (12) and iFoldRNA (13); however, they do not permit folding of RNA molecules composed of multiple chains or with complex restraints, such as utilizing a pre-defined 3D structure.

We have recently introduced SimRNA, a method for RNA folding simulation and 3D structure prediction that uses a coarse-grained representation of five atoms per residue and a statistical potential methodology (14). It can predict RNA 3D structure from sequence alone, and, if available, can use additional structural information in the form of secondary structure restraints, distance restraints that define the local arrangement of certain atoms, and can jump-start the simulation with a 3D structure provided in a PDB file. Thus far, SimRNA has been available as a stand-alone package that required the user to have good computer skills and a powerful computer. Here, we introduce a web service that simplifies the steps of the stand-alone package, does not require the user to supply computing power and memory, provides a simple interface for the user, and displays the progress of the simulation in real time. This renders the approach available to an individual who is not necessarily an expert in RNA structure and does not have access to state-of-the-art 3D molecular modeling facilities, but who needs a model of the RNA 3D structure, for instance to design biochemical experiments, or may want to observe the conformational changes of the RNA as it folds.

## METHODS

### Workflow implemented as SimRNAweb

**Figure 1.**
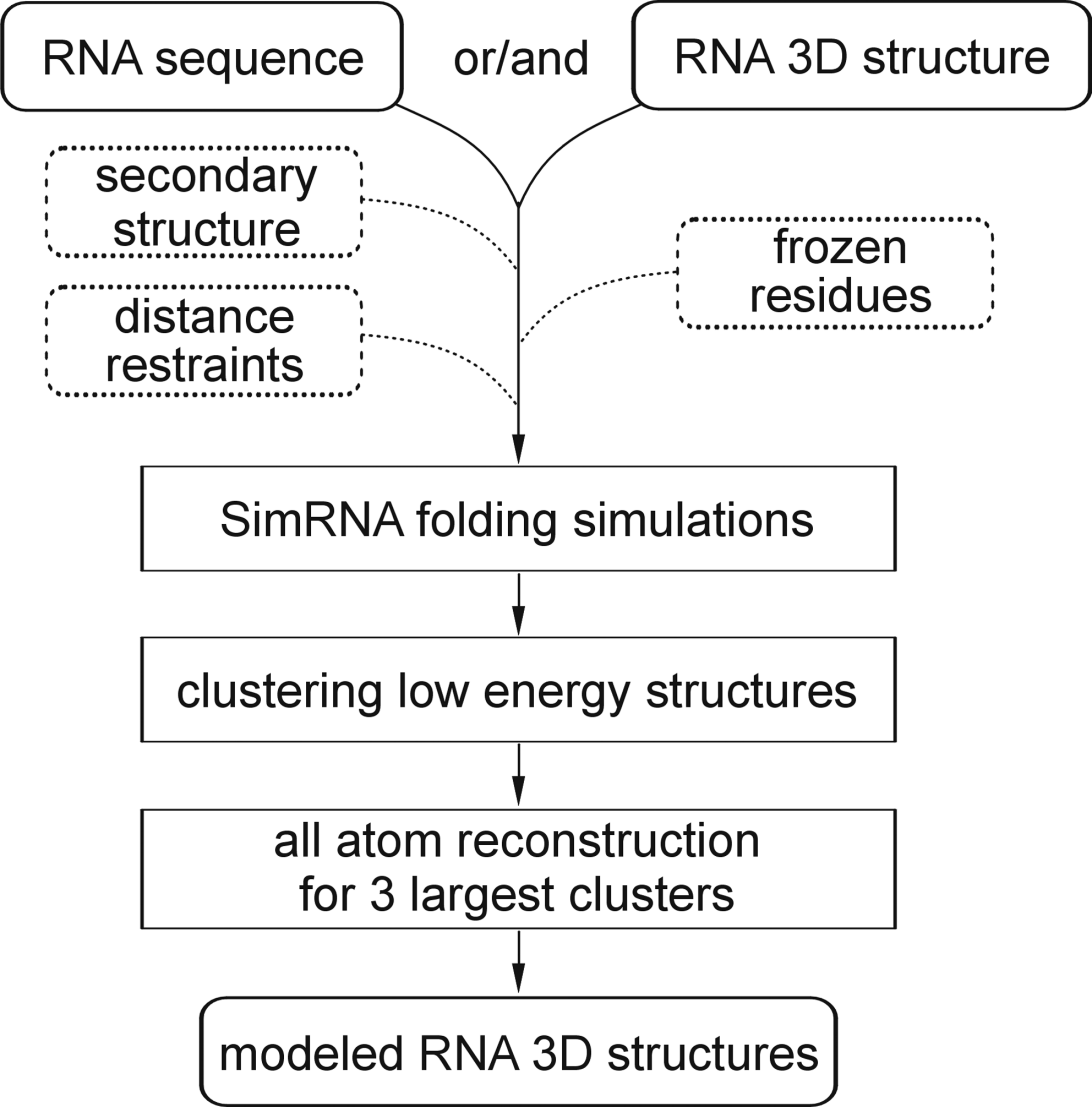
The workflow of the SimRNAweb server.

The primary purpose of SimRNAweb is to carry out all the typical tasks normally required in a SimRNA calculation and to provide the computer resource needed to obtain an output structure. However, SimRNAweb also provides special features such as showing the progress of the simulation and showing the structure during the progression of the simulation. During the simulation and at the end, the best-scored conformations are clustered and the all-atom models of representatives of the three largest clusters are generated. In the output, structures of these three models are displayed using JSmol (15), and their coordinates are made available for download. Therefore, the user does not have to carry out the tasks of a simulation, processing the resulting files, clustering the results, and converting the data from a five-atom representation to an all atom representation—all this is done remotely by the server (Figure 1). Thus, even though SimRNA uses a coarse-grained representation for the folding simulation, the user can submit PDB-formatted files in all atom or SimRNA representation (files containing only the atoms used by SimRNA) and receives the results as all atom PDB-formatted files. All log files and intermediate data are also made available to users who need them for more advanced analyses or who are simply curious about all the details of the calculations (in SimRNA representation).

### Required input files—sequence and/or 3D structure

The smallest set of input data consists of an RNA sequence in a single line. Alternatively, a PDB structure in the PDB format can be submitted. Starting a simulation from a pre-defined 3D structure can be used in a variety of applications, such as sampling the conformational space in a vicinity of a given conformation, testing the ability of a given RNA sequence to maintain different 3D architectures or refining a structural model built with some other technique such as comparative/homology modeling.

Regardless of whether the user submits a sequence or a PDB file, in a separate window, the user can supplement the input information with RNA secondary structure, additional information such as a file containing distance restraints between the atoms. In the case of submitting a PDB file, the user can specify positions in the 3D structure that need to be frozen (kept rigid) in the course of the simulation. All of this information can be submitted simultaneously. If a PDB file is supplied, then all the labels should be ATOM and any non-RNA species should be removed from the file. SimRNA relies only on five atoms per nucleotide: P, C4′, N1, C2, C4 (for pyrimidine bases) or P, C4′, N9, C2, C6 (for purine bases). All other atoms are ignored (and don't need to be removed). As long as these five basic atoms are present for a given residue of RNA, SimRNA can process the information. SimRNA *can* directly read HETATM tags, but the name of the residue and the five atoms mentioned above must be set to standard residues (A, C, G or U). In principle, SimRNA can read PDB files with chemically modified residues and interpret them as A, C, G or U, but this requires manual editing of the residue and atom names and we recommend this feature to expert users only. Please refer to the online manual for more detailed information.

Many PDB files have missing terminal and internal residues and the sequence of residues inferred from the coordinates in the PDB file is not always identical in every respect to the sequence that should be modeled. When an incomplete PDB file is submitted to SimRNAweb as a starting structure, a complete sequence can also be submitted to fill in missing spaces and model small insertions. When submitting a PDB file together with a sequence, SimRNAweb will automatically try to align the submitted sequence with the sequence in the PDB file, according to the numbering of residues in the PDB file. The alignment rules are currently absolutely strict: the *n*th residue in sequence must correspond to residue number *n* in the PDB file; and it is the responsibility of the user to provide structures and sequences that correspond in residue identity and number. Any submission in which the input structure is different than or misaligned with the input sequence may be processed erroneously. It should be also emphasized that many PDB files have arbitrary numbering of residues that does not correspond to the actual sequence; e.g. the first residue in the PDB file can have an index smaller or larger than 1 (in the case of engineered molecules or sequence fragments), while the first residue in sequence always has an index of 1. Hence, the combined input of sequence and structure is a preliminary, experimental feature and should be used with caution. We recommend it only to advanced users who can edit PDB files with third-party programs of their choice. At this stage SimRNAweb does not provide any support for inferring, verifying, or correcting alignments between the input sequence and structure files, and it does not support homology modeling (replacement of one sequence in the input file with another sequence). For such operations we recommend the ModeRNA server (16).

### Secondary structure restraints

When information on the secondary structure (including pseudoknots) is available, it can be provided as restraints. When only secondary structure is involved, this can be entered as a single line in a dot bracket format and for pseudoknots, multiple lines demarking overarching helices should be used (see the online documentation).

### Distance restraints

Distance restraints can represent any type of pairwise interaction as long as it can be defined in terms of any pair of the five atoms (P, C4′ and N1, C2, C4 for pyrimidines or N9, C2, C6 for purines) or a virtual point at the middle of the base called MB. Distance restraints come in two forms: SLOPE and WELL. A typical SLOPE restraint has the form ‘SLOPE A/23/C4′ C/45/P 5.5 8.5 0.2’, where the last number indicates a penalty in SimRNA energy units (these units do not correspond to standard energy units; they depend on the statistical force field). It says that atom C4′ of residue 23 on chain A should be between 5.5 and 8.5 Å to atom P of residue 45 of chain C to avoid an energy penalty, and when this distance is <5.5 Å or >8.5 Å, the penalty increases by 0.2 unit of energy per Å (so at a distance 10.5 or 3.5 Å, the penalty will be 0.4 units). A typical WELL restraint has the form ‘WELL A/23/C4′ C/45/P 6.5 7.5 0.5’, where the last number indicates a reward in SimRNA energy units (depth of the well). It says that the structure should essentially fall into a ‘well’ that has -0.5 units of energy when the atoms A/23/C4′ and C/45/P are between 6.5 and 7.5 Å, and outside of this region there is no effect to the energy. Together, these two restraints are intended to encourage this pair of atoms (C4′ and P) to locate themselves at ∼7 Å from each other: irrespective of anything else we know about chains A or C. In general, restraints should be used to encourage the structure to form in a certain way. We recommend using ranges of distance to represent the uncertainty of the distance, and the energy units as a relative weight of restraints. Further explanation can be found in the online manual.

### PDB restraints

A separate field in the submission form for the server permits the user to specify which parts of the structure are frozen by writing the chain and the frozen residues, e.g. ‘A:1–10, 15–20; B:1–10’, where A and B are the chains and the residue indices should be numbered according to the PDB file. This feature can be used to keep some parts of the model that should be left unchanged and refold other parts, with or without other restraints.

### Parameters of the RNA folding simulation

In addition to the main input files, the user can reduce the simulation length from 500 frames (default value) to a shorter run. Particularly when restraints are used, the run is typically solved within 300 frames for a sequence of up to 75 nucleotides in length. Other options are to cut the simulation in the middle of a run, specify different parts of the PDB structure to free and parts of the PDB structure to join with an input sequence.

### Training (parametrization) and testing datasets

The structure prediction engine and the default parameters of the simulation are currently (February 2016) identical to those of the published version of SimRNA version 3.20 (14). Only one minor correction has been introduced to define the set of atoms frozen if a residue is specified as frozen. SimRNA v.3.20/1 was benchmarked against a variety of test structures including the dataset from Ding *et al*. (17), the 2008 Das&Baker dataset (18), the 2010 Das&Baker dataset of RNA structural motifs (19), the Seetin&Mathews dataset (20) and published structures available from RNA puzzles (21,22). With the exception of the Das&Baker motifs dataset, which required restraints on the bordering parts of the crystal structure to do the study, all the sequences were tested both in *de novo* simulations and using various levels of restraints including secondary structure and distance restraints. According to these benchmarks, SimRNA is competitive with other methods, including FARNA and iFoldRNA. It often predicts 3D structures for RNA molecules up to 100 nt correctly, and secondary structure and tertiary restraints improve these predictions and allow for folding much longer sequences.

## RESULTS

### SimRNAweb server

The SimRNAweb server is an automated and user-friendly implementation of SimRNA, a method for RNA 3D structure modeling developed in our laboratory (14) and used in practice in many cases, including participation in the RNA Puzzles experiment (21,22). SimRNAweb server has been running since September 2015. So far, it has processed several hundred predictions (folding *de novo* and under restraints) and an increasing number of individuals outside our group have been involved in running and testing. Most recently, SimRNAweb has entered the new category of fully automated RNA 3D structure prediction of the RNA Puzzles competition.

### Example applications

**Figure 2.**
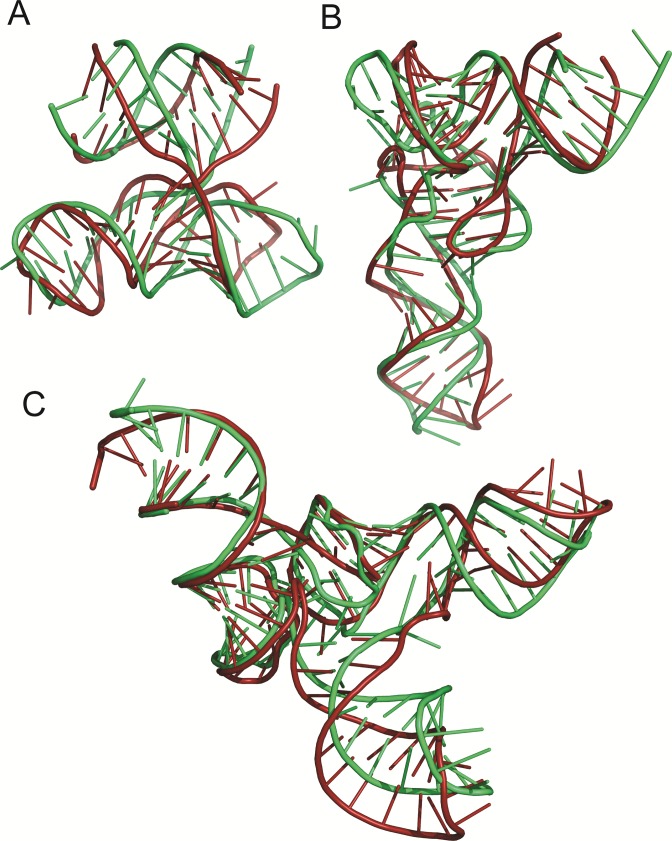
Examples of RNA 3D structure prediction with SimRNAweb. Green indicates the reference structure (PDB ids: **A** – 1kh6, **B** – 3l0u, **C** – 3w3s), red indicates models generated with SimRNAweb (representatives of the first cluster).

Figure 2A illustrates an example of RNA 3D structure prediction with SimRNAweb, based on sequence information alone (without secondary structure or any other restraints). An experimentally determined molecule of RNA tertiary domain essential to HCV IRES-mediated translation initiation comprises two chains from the crystal structure (PDB id.: 1kh6). SimRNAweb run with default parameters folded it to a model that matched the reference structure very well (correct secondary structure, correct orientation of all structural elements, and RMSD of 5.9 Å). The additional application of secondary structure restraints improves this model to 4.6 Å (data not shown).

Figure 2B shows a predicted 3D structure for tRNA(Phe) from *Escherichia coli* (73 residues), for an input comprising sequence, secondary structure, and distance restraints. If only secondary structure restraints are used, the resulting model approaches RMSD of 12 Å to the crystal structure. However, when known tertiary interactions between residues 8–14 and 48–50 are accounted for with just six pairwise distance restraints, the server generates a model with a native-like topology and local contacts, and with RMSD of 6.1 Å.

Finally, Figure 2C shows the results of refining a homology model using the SimRNAweb server. Here, the starting structure of *Aquifex aeolicus* tRNA(Sec) (99 residues) was homology-modeled based on the crystal structure of *A. aeolicus* tRNA(Met) using the ModeRNA server (16). Confidently modeled regions, including the tRNA core, were frozen, while the loops and the extra arm (not present in the template) were allowed to refold. Comparison of the starting structure and the locally refolded model with the experimentally determined structure of *A. aeolicus* tRNA(Sec) reveals an improvement in RMSD from 5.6 to 4.1 Å RMSD in the process.

## DISCUSSION

SimRNAweb offers a new user-friendly interface for the SimRNA program, delivering RNA 3D structure modeling to any user. The server accepts an RNA sequence and can enable folding with a mixture of various types of restraints including secondary and/or 3D structure and/or distance restraints. The user can also submit sequences or structures containing multiple RNA chains (up to 52) and thereby SimRNAweb can be used for studying RNA-RNA interactions. Starting a simulation from a pre-defined 3D structure can be used in a variety of application, such as refinement of models built with other methods, sampling the conformational space in a vicinity of a given conformation, and testing the ability of a given RNA sequence to maintain different 3D architectures. Advanced applications of SimRNAweb, which make use of folding with restraints, include docking pre-folded RNA molecules/domains/motifs to each other, and optimizing homology models, with confident parts of the structures completely frozen or highly restrained, and with other regions allowed to change conformation upon folding and/or binding.

## AVAILABILITY

The web server is available at http://genesilico.pl/SimRNAweb. This website is free and open to all users and there is no login requirement.
